# Electrocardiographic and echocardiographic evaluation in dogs with hypothyroidism before and after levothyroxine supplementation: A prospective controlled study

**DOI:** 10.1111/jvim.15600

**Published:** 2019-08-29

**Authors:** Carlo Guglielmini, Michele Berlanda, Federico Fracassi, Helen Poser, Shani Koren, Marco Baron Toaldo

**Affiliations:** ^1^ The Department of Animal Medicine, Production and Health University of Padua Padua Italy; ^2^ The Department of Veterinary Medical Sciences Alma Mater Studiorum—University of Bologna Bologna Italy

**Keywords:** canine, echocardiography, electrocardiography, endocrine disease

## Abstract

**Background:**

Improvement in cardiac function has been demonstrated after thyroxine treatment in humans with hypothyroidism using the myocardial performance index (MPI). Cardiac changes after thyroxine supplementation are poorly documented in dogs with spontaneous hypothyroidism and comparison with clinically healthy dogs is lacking.

**Objectives:**

To evaluate the electrical activity and mechanical function of the heart in dogs with primary hypothyroidism at baseline (T0) and after thyroxine supplementation (T60).

**Animals:**

Forty client‐owned dogs with hypothyroidism and 20 clinically healthy dogs.

**Methods:**

Prospective cohort study. Selected electrocardiographic and echocardiographic variables, including the MPI, were measured in all dogs at T0 and in 30 hypothyroid dogs at T60.

**Results:**

Hypothyroid dogs had significantly decreased median or mean heart rate (HR), P wave amplitude, and R wave amplitude (*P* = .04, *P* = .002, and *P* = .003, respectively) and E‐point‐to‐septal separation normalized to body weight (EPSSn) and trans‐mitral E wave velocity (E max; *P* < .001 and *P* = .025, respectively) at T0 compared to control dogs. At T60, significantly increased median or mean HR, P wave amplitude, fractional shortening, and E max (*P* < .001, *P* = .004, *P* = .002, and *P* = .009, respectively) and significantly decreased left ventricular end‐diastolic volume index, and normalized systolic diameter and EPSSn (*P* = .03, *P* = .03, and *P* = .001, respectively) were found.

**Conclusions and Clinical Importance:**

Hypothyroidism in dogs induces mild and reversible changes of electromechanical cardiac function. The MPI does not have clinical importance in identifying cardiac dysfunction in affected dogs.

**Abbreviations**
2D: 2‐dimensional
Ao: aortic root diameter
bpm: beats per minute
BW: body weight
cTSH: canine thyrotropin hormone
DCM: dilated cardiomyopathy
EF: ejection fraction
EPSS: E point‐to‐septal separation
EPSSn: E point‐to‐septal separation normalized for body weight
FS: fractional shortening
HR: heart rate
LA: left atrial diameter
LA/Ao: left atrium to aortic root diameter ratio
LVDD: left ventricular diastolic diameter
LVDDn: left ventricular diastolic diameter normalized for body weight
LVDS: left ventricular diameter in systole
LVDSn: left ventricular diameter in systole normalized for body weight
MEA: mean electrical axis
MMVD: myxomatous mitral valve disease
MPI: myocardial performance index
rhTSH: recombinant human TSH
TT4: total thyroxine

## 1 INTRODUCTION

Acquired primary hypothyroidism is a common thyroid disorder in dogs that may cause decreased metabolic rate and dysfunction of almost every organ in the body. The most commonly reported clinical manifestations of primary hypothyroidism in dogs include dermatologic signs as well as reproductive, neurological, and cardiovascular abnormalities.^1^, ^2^ In particular, retrospective clinical and experimental studies reported changes of both cardiac electrical and mechanical functions in dogs with spontaneous and induced thyroid hormone deficiency.^3^, ^4^, ^5^, ^6^, ^7^, ^8^, ^9^, ^10^, ^11^, ^12^, ^13^ Reported ECG abnormalities in hypothyroid dogs include cardiac arrhythmias, such as sinus bradycardia,^7^, ^8^ atrial fibrillation,^4^, ^5^, ^6^, ^10^ and atrioventricular blocks,^7^, ^9^, ^12^, ^14^ decreased R wave amplitude,^9^, ^10^, ^12^, ^14^ and decreased amplitude or inverted T wave.^10^ Partial response to thyroxine treatment with normalization of ECG changes was reported in some dogs,^6^, ^7^, ^8^, ^9^, ^10^ and conversion of atrial fibrillation to sinus rhythm was observed in a dog treated solely with thyroxine.^3^

Previous echocardiographic studies in dogs with hypothyroidism have been predominantly focused on evaluation of left ventricular (LV) systolic cardiac function. Echocardiographic changes observed in dogs with spontaneous or experimentally induced hypothyroidism include increased left ventricular diameter in systole (LVDS) and diastole (LVDD),^5^, ^9^ decreased LV fractional shortening (FS),^3^, ^5^ prolonged pre‐ejection period, and decreased velocity of circumferential fiber shortening.^9^ All of these alterations might mimic a familiar form of primary cardiomyopathy such as dilated cardiomyopathy (DCM).^15^ Thus, it was previously thought that hypothyroidism‐induced cardiomyopathy should be considered a differential diagnosis for DCM in dogs.^15^ However, LV systolic function and time intervals should improve in dogs with hypothyroid‐induced cardiac changes when appropriate hormone replacement treatment has been established.^16^

In humans, both systolic and diastolic cardiac function can be influenced by thyroid hormones,^17^ and the myocardial performance index (MPI), defined as the sum of isovolumetric contraction time plus relaxation time divided by ejection time, has been proposed as a useful marker of combined systolic and diastolic cardiac function.^18^, ^19^ Measurement of MPI in human patients with hypothyroidism showed deterioration of cardiac function that was reversible after treatment with thyroxine.^17^, ^20^ No study has prospectively investigated the effects of naturally occurring hypothyroidism on both systolic and diastolic function using the MPI in dogs. Furthermore, previous studies on cardiac dysfunction in dogs with hypothyroidism were carried out without the use of a control group of clinically healthy dogs. Thus, we evaluated cardiac electrical activity and mechanical function, including measurement of MPI, in euthyroid dogs and dogs with primary hypothyroidism before and after thyroxine supplementation.

## 2 MATERIALS AND METHODS

### 2.1 Animals

Client‐owned dogs presented to 2 veterinary teaching hospitals and with a final diagnosis of primary hypothyroidism were prospectively included in the study. After the owner signed a written consent form, each dog underwent physical examination, ECG, echocardiographic examination, and blood sampling for CBC and serum biochemical profile. Dogs were considered hypothyroid if they showed consistent clinical signs of hypothyroidism (eg, dermatological abnormalities, lethargy, asthenia, exercise intolerance, cold intolerance, obesity, weight gain), serum total thyroxine (TT4), and canine thyrotropin hormone (cTSH) concentrations below and above the reference range, respectively (ie, TT4 <0.93 μg/dL and cTSH >0.5 ng/mL). In dogs with serum TT4 concentration below the reference range and normal serum cTSH concentration, a recombinant human TSH (rhTSH; Thyrogen, Genzyme Corp, Haverhill, Suffolk, UK) stimulation test was performed. These dogs were enrolled if both the prestimulation and poststimulation TT4 concentration were below the reference range (ie, TT4 <0.93 μg/dL). Dogs that had received thyroid hormone replacement treatment within the previous 8 weeks were excluded. Dogs with concurrent endocrine disorders (eg, diabetes mellitus, hyperadrenocorticism, hypoadrenocorticism), chronic renal failure, or chronic hepatic failure also were excluded.

After cardiac evaluation, the hypothyroid dogs were started on treatment with levothyroxine PO as a liquid formulation (Leventa; Intervet Inc, Millsboro, Delaware; starting dosage 20 μg/kg q24h) or tablets (Canitroid; Eurovet Animal Health BV, Handelsweg, The Netherlands; starting dosage 10‐15 μg/kg q12h). Reevaluations were performed at approximately 30 and 60 days from treatment initiation and included clinical history, physical examination, and evaluation of serum TT4 concentration, measured 4‐6 hours after administration of levothyroxine. If necessary, the levothyroxine dose then was adjusted. Treatment was considered appropriate if serum TT4 concentration was within or mildly above the reference range. At the time of reevaluation, after approximately 2 months of treatment (T60), cardiac evaluation was repeated, assuming that thyroid hormone replace treatment was appropriate.

A control population of 20 clinically healthy dogs also was enrolled. They were recruited from the patients of the 2 hospitals presented for periodic routine visits or for evaluation before an elective procedure. Dogs were considered healthy on the basis of clinical history and normal results of physical examination, CBC, and serum biochemical profile, including serum TT4 and cTSH concentrations (ie, serum TT4 and cTSH >0.93 μg/dL and <0.5 mg/mL, respectively). Moreover, these animals were considered to have normal cardiovascular health if results of echocardiographic examination were within normal limits and no rhythm disturbance (other than sinus arrhythmia or sinus tachycardia) was observed on ECG.

### 2.2 Analytical procedures and thyroid evaluation

Serum cTSH concentrations were measured by use of a solid‐part, 2‐site chemiluminescent enzyme immunometric assay (IMMULITE 1000 Canine TSH; Diagnostic Products Corporation, Los Angeles, California).^21^ The intra‐assay coefficients of variation were 5.0%, 4%, and 3.8% at TSH concentrations of 0.20, 0.50, and 2.6 ng/mL, respectively. The interassay coefficients of variation were 6.3% and 8.2% at TSH concentrations of 0.16 and 2.8 ng/mL, respectively. The sensitivity of the assay was 0.03 ng/mL. The upper limit of the reference range was 0.5 ng/mL. Serum TT4 concentrations were determined using a homologous solid‐phase, chemiluminescent enzyme immunoassay (IMMULITE 1000 Canine total T4, Diagnostic Products Corporation). The intra‐assay and interassay coefficients of variation were 3.9%‐10.8% and 5.2%‐13.8%, respectively. The sensitivity of the assay was 0.5 μg/dL. The reference range for TT4 was 0.93‐2.87 μg/dL. To perform the rhTSH‐stimulation test, serum TT4 was measured before and 6 hours after the IV administration of recombinant human TSHc at a dose of 75 μg per dog.^22^

### 2.3 Cardiac evaluation

A complete cardiac evaluation including physical examination, 6 lead standard ECG, and trans‐thoracic 2‐dimensional (2D), M‐mode, and echo‐Doppler echocardiogram was performed at T0 in all dogs and at T60 in hypothyroid dogs.

Each ECG examination was performed with the dogs placed in the right lateral recumbency using dedicated devices (Archiwin, Esaote S.p.A., Firenze, Italy; Cube ECG, Cardioline S.p.A., Caverano, Italy; TouchECG HD, Cardioline S.p.A). Two‐minute recordings were acquired to detect the presence of rhythm disturbances. Electrocardiographic measurements were performed by analyzing a 10‐second strip, using a ruler. Intervals and wave amplitude were obtained as multiple of 10 ms and 0.1 mV, respectively. Variables analyzed included cardiac rhythm (eg, normal sinus rhythm, sinus arrhythmia, pathological arrhythmias), heart rate (HR) in beats per minute (bpm) calculated by determining the number of QRS complexes in a 3‐second interval and multiplying this number by 20 (reference range, 60‐160 bpm),^23^ amplitude and duration of the P wave, PQ‐interval duration, R wave amplitude and duration of the QRS complex, duration of the QT interval corrected for HR according to the logarithmic formula (QT interval corrected = log600 **×** QT/logRR),^24^ and mean electrical axis (MEA) of the QRS complex calculated using the following equation: MEA = arctan (Iamp, aVFamp) **×** 180/π.^25^

Echocardiographic images were obtained in awake animals using ultrasound units (CX50, Philips, Eindhoven, The Netherlands; iE33 ultrasound system, Philips Healthcare, Monza, Italy) equipped with 1‐5 or 3‐8 MHz phased‐array transducers and simultaneous ECG trace recording, according to previously published standards.^26^ An M‐mode interrogation of the LV was performed from the right parasternal short‐axis view at the level of the chordae tendineae, and measurements were obtained using the leading edge‐to‐leading edge method.^27^

The LVDD and LVDS were indexed to the body weight (BW) according to Cornell's method^28^ to obtain their normalized measurements (LVDDn and LVDSn, respectively). The LV FS was calculated according to the formula: FS = [(LVDD − LVDS)/LVDD] × 100. The mitral valve E point‐to‐septal separation (EPSS) was measured from M‐mode images obtained from the short‐axis view at the level of the mitral valve, as previously described in dogs.^29^ Because EPSS is a linear dimension, mildly influenced by the weight of the animal, the absolute value then was indexed to BW (EPSSn) using the formula: EPSSn = (EPSS/BW^**1/3**^).^28^, ^30^ Left atrial (LA) and aortic root (Ao) diameters were measured using a 2D method from the right parasternal short‐axis view at the heart base level in early diastole, and their ratio (LA/Ao) was calculated.^31^ End‐diastolic and end‐systolic volumes of the LV were calculated from images obtained from the right parasternal 4‐chamber long‐axis view by the Simpson method of discs and then indexed to body surface area. The LV ejection fraction (EF) was obtained using the equation: EF = ([end‐diastolic volume − end‐systolic volume]/end‐diastolic volume) × 100.

Aortic flow was interrogated by pulsed‐wave Doppler from the subcostal window and peak velocity was recorded. Transmitral flow was interrogated from the left apical 4‐chamber view and peak velocities of the early diastolic (E) and late diastolic (A) waves were recorded. Spectral Doppler interrogation of LV inflow and outflow tracings was obtained from a left apical 5‐chamber view and the systolic times were measured. The LV MPI was calculated according to the formula: MPI = (isovolumetric contraction time + isovolumetric relaxation time)/LV ejection time.^32^

Each echocardiographic and echo‐Doppler variable was measured 3 times, and the averaged value was used for statistical analysis.

Dogs enrolled in the control group were considered to have a normal echocardiographic examination if no clinically relevant valvular insufficiency was noted on color Doppler examination, no congenital abnormalities or signs of pulmonary hypertension were detected, and LVDDn, LVDSn, and LA/Ao were within normal ranges (ie, ≤1.8, <1.3 and <1.6, respectively).^28^, ^31^

Each ECG and echocardiographic examination was reviewed by a board‐certified cardiologist (M.B.T.) who performed all of the measurements.

### 2.4 Statistical analysis

All data were tested for their distribution using a D'Agostino‐Pearson normality test. Normally distributed data are reported as mean ± standard deviation, and nonnormally distributed data are reported as median and range (minimum and maximum).

Continuous variables were compared between hypothyroid and control dogs using an unpaired Student's *t* test or a Mann‐Whitney *U* test, whereas comparison between hypothyroid dogs before and after treatment was carried out using a paired Student's *t* test or a Wilcoxon signed rank test. Descriptive categorical data were compared using a Fisher's exact test.

Statistical analysis was conducted using commercially available programs (Microsoft Excel; Microsoft Office 2011, Microsoft Corporation, Bellevue, Washington; Prism 5, GraphPad Software Inc, San Diego, California). A value of *P* < .05 was considered significant.

## 3 RESULTS

### 3.1 Animals

Forty recently diagnosed hypothyroid dogs were recruited. Breeds included 13 mixed breeds, 3 Maremma Sheepdogs and Irish Setters, 2 Italian Hounds, Labrador Retrievers, Doberman Pinschers, and Lagotto Romagnolo, and 13 dogs of other breeds represented by 1 dog each. There were 19 intact males and 21 females (11/21 were spayed females), with a median age of 5.1 years (range, 3‐10 years) and BW of 28 ± 13 kg. The control group of healthy dogs included 7 mixed breeds, 2 Labrador Retrievers, Golden Retrievers, Cocker Spaniels, and American Staffordshire Terriers, and 4 dogs of other breeds represented by 1 dog each. This control group included 7 males (2/7 were castrated males) and 13 females (4/13 were spayed females) with a median age of 5.4 years (range, 2‐14 years) and BW of 26 ± 8 kg. No significant differences in distribution of sex, age, and BW were observed between the 2 groups. The most common clinical signs in the hypothyroid dogs were dermatological abnormalities (74%), asthenia or exercise intolerance (56%), lethargy (59%), depression (54%), and weight gain (49%). At the time of diagnosis, the median hematocrit was 37.5% (range, 28.5‐59.6%), and the hematocrit was mildly below the reference range (37.0‐55.0%) in 16 of 40 dogs (40%). The mean cholesterol concentration was 529 ± 252 mg/dL, and 27 of 40 dogs (67%) had cholesterol concentrations above the reference range (140‐350 mg/dL). The median serum concentration of TT4 in hypothyroid dogs was 0.5 μg/dL (range, 0.5‐0.7 μg/dL) because many dogs had TT4 concentration at the lower limit of detection of the test. Their median cTSH concentration was 0.78 ng/mL (range, 0.06‐11.9 ng/mL), and 34 (85%) of these dogs had increased serum cTSH concentration. The rhTSH stimulation test was performed on 13 of 40 hypothyroid dogs (32.5%). The median pre‐stimulation and post‐stimulation TT4 concentrations of these dogs were 0.5 μg/dL (range, 0.5‐0.7 μg/dL) and 0.5 μg/dL (range, 0.5‐0.54 μg/dL), respectively. Table 1 presents clinical and baseline hormonal data.

**Table 1:** Descriptive data obtained from 60 dogs with (hypothyroid T0) and without (control g) hypothyroidism

Variable	Hypothyroid T0	Control group	*P* value
Number of dogs	40	20	NA
Sex (male/female)	19/21	7/13	.42
Age (y)	5.1 (3–10)	5.4 (2–14)	.13
Body weight (kg)	28 ± 13	26 ± 8	.60
TT4 (μg/dL)	0.5 (0.5–0.7)	1.3 (1‐2.3)	<.001
cTSH (ng/mL)	0.78 (0.06‐11.9)	0.13 (0.03‐0.48)	<.001

*Note:* Normally and nonnormally distributed data are expressed as mean ± standard deviation and median and range (minimum–maximum), respectively.

Abbreviations: cTSH, canine thyrotrophin hormone; TT4, serum total thyroxine.

Thirty of the 40 hypothyroid dogs (75%) from the initial study group completed the study and were reevaluated (T60) after a median time of 70 days (range, 49‐166 days) from the beginning of levothyroxine supplementation. Of the remaining 10 animals, 9 dogs were lost to follow‐up and 1 dog died spontaneously with clinical signs of intracranial disease. At reevaluation at T60, all dogs showed general improvement of clinical signs. Almost all dogs showed improvement in general metabolic signs, including 21 of 22 dogs, 23 of 23 dogs, and 20 of 21 dogs showing asthenia, lethargy, and depression, respectively; 20 of 39 dogs (51%) showed a decrease in BW; and 22 of 29 dogs with dermatologic abnormalities (76%) showed complete resolution or marked improvement of skin and hair coat alterations. The median TT4 concentration at T60 was 3.19 μg/dL (range, 0.93‐9.71 μg/dL). In 21 of 30 dogs (70%), cTSH concentration was reevaluated and median cTSH concentration was 0.11 ng/mL (range, 0.03‐0.38 ng/mL). Eighteen of these 21 dogs (86%) had high cTSH concentrations at T0, which returned into the reference range at the time of reevaluation; the other 3 of 21 dogs (14%) had cTSH concentrations within the reference range at T0 that remained within the reference range at the time of reevaluation.

### 3.2 ECG and echocardiographic data

Electrocardiographic and echocardiographic data at T0 of control dogs and dogs with hypothyroidism are summarized in Table 2. Regarding the ECG variables, only 1 hypothyroid dog (2.5%) and 1 control dog had HR <60 bpm and sinus tachycardia (ie, HR = 200 bpm), respectively, without other cardiac arrhythmias. One hypothyroid dog had complete right bundle branch block with QRS complex duration of 100 milliseconds and MEA of the QRS of −99° that persisted at T60. The median HR of hypothyroid dogs (80 bpm; range, 50‐160 bpm) was significantly lower compared to that of control dogs (100 bpm; range, 80‐200 bpm; *P* = .04). Furthermore, hypothyroid dogs had significantly lower median P‐wave amplitude (0.15 mV; range, 0.05‐0.35 mV) and mean R wave amplitude (1.00 ± 0.45 mV), and significantly different median MEA QRS (61°; range, −99 − +96°) compared to those of control dogs (0.20 mV; range, 0.10‐0.40 mV, *P* = .002; 1.48 ± 0.76 mV, *P* = .003; 79°; range, 45°‐90°; *P* < .001, respectively). Regarding echocardiographic variables, the median EPSSn (2.29; range, 0.48‐7.1) was significantly higher in hypothyroid dogs compared to that of control dogs (1.27; range, 0.41‐2.32; *P* < .001). Furthermore, a significantly lower mean E wave velocity (60 ± 15 cm/s) was found in hypothyroid dogs compared to that of control dogs (70 ± 18 cm/s; *P* = .02).

**Table 2:** Electrocardiographic and echocardiographic variables from 60 dogs with (hypothyroid T0) and without (control group) hypothyroidism at the time of diagnosis

	Hypothyroid T0	Control group	
Variable	(n = 40)	(n = 20)	*P* value
Electrocardiography			
HR (bpm)	**80 (50–160)**	**100 (80–200)**	**.04**
P‐wave amplitude (mV)	**0.15 (0.05–0.35)**	**0.20 (0.10‐0.40)**	**.002**
P‐wave duration (ms)	40 (30‐50)	40 (30‐50)	.91
PQ (ms)	100 (70‐140)	100 (40‐130)	.98
QRS (ms)	60 (30‐100)	70 (60‐81)	.10
R‐wave amplitude (mV)	**1 ± 0.45**	**1.48 ± 0.76**	**.003**
MEA QRS (°)	**61 (−99 to 96)**	**79 (45‐90)**	**<.001**
QTc (ms)	201 ± 18	203 ± 12	.80
Echocardiography			
LA/Ao	1.3 (1.0‐2.6)	1.3 (0.9‐1.5)	.63
LVDDn	1.5 ± 0.2	1.5 ± 0.1	.59
LVDSn	1.1 (0.6‐1.6)	1.1 (0.8‐1.2)	.93
FS (%)	26 ± 9	25 ± 7	.66
EPSSn	**2.29 (0.48‐7.1)**	**1.27 (0.41‐2.32)**	**<.001**
E‐wave velocity (cm/s)	**60 ± 15**	**70 ± 18**	**.02**
A‐wave velocity (cm/s)	49 ± 16	54 ± 14	.21
MPI	0.57 ± 0.23	0.54 ± 0.20	.63
EDVi (mL/m^2^)	65 (28‐101)	71 (42‐88)	.99
ESVi (mL/m^2^)	36 ± 13	33 ± 9	.44
EF (%)	48 ± 11	50 ± 13	.51

*Note:* Normally and not normally distributed data are expressed as mean ± standard deviation and median and range (minimum–maximum), respectively. Values with statistically significant difference are indicated in bold.

Abbreviations: EDVi, end diastolic volume indexed to body surface area; EF, ejection fraction; EPSSn, E‐point‐to‐septal separation normalized to body weight; ESVi, end systolic volume indexed to body surface area; FS, fractional shortening; HR, heart rate; LA/Ao, left atrial to aortic ratio; LVDDn, left ventricular diastolic diameter normalized to body weight; LVDSn, left ventricular systolic diameter normalized to body weight; MEA, mean electrical axis; MPI, myocardial performance index; QTc, QT interval corrected for heart rate.

Comparisons between ECG and echocardiographic variables obtained at T0 and T60 in 30 hypothyroid dogs are summarized in Table 3. After supplementation with levothyroxine, dogs with hypothyroidism showed significantly increased HR (*P* < .001) and P‐wave amplitude (*P* = .009) on ECG tracings, significantly decreased LVDSn (*P* = .04) and EPSSn (*P* = .001), and significantly increased FS (*P* < .001) and E‐ and A‐wave velocity (*P* = .02, and *P* = .003, respectively) on echocardiography. Regarding echocardiographic indices of LA and LV dimension, 5 hypothyroid dogs had LA/Ao ≥1.6 at T0 that remained increased in only 2 of them at T60; 5 dogs had LVDDn at the upper limit of normality (1.8) at T0, but all dogs had a LVDDn ≤1.7 at T60; and, 2 dogs had LVDSn of 1.4 at T0 and only 1 dog had LVDSn equal to 1.3 at T60. The MPI did not differ between control dogs (0.54 ± 0.20) and hypothyroid dogs at T0 (0.57 ± 0.23; *P* = .63) and between 30 hypothyroid dogs at T0 (0.56 ± 0.24) and T60 (0.55 ± 0.16; *P* = .82).

**Table 3:** Electrocardiographic and echocardiographic variables from 30 dogs with hypothyroidism before (hypothyroid T0) and after treatment with levothyroxine (hypothyroid T60)

Variable	Hypothyroid T0	Hypothyroid T60	*P* value
Electrocardiography			
HR (bpm)	**80 (50–160)**	**120 (67‐180)**	**<.001**
P‐wave amplitude (mV)	**0.15 (0.05‐0.35)**	**0.20 (0.10‐0.45)**	**.004**
P‐wave duration (ms)	40 (30‐50)	40 (0.15‐50)	.09
PQ (ms)	100 (80‐140)	98 (30‐160)	.06
QRS (ms)	63 (30–100)	65 (40‐90)	.67
R‐wave amplitude (mV)	1.04 ± 0.48	1.13 ± 0.51	.33
MEA QRS (°)	60 (−99‐90)	63 (−99‐90)	.93
QTc (ms)	204 ± 19	204 ± 16	.98
Echocardiography			
LA/Ao	1.3 (1‐2.6)	1.22 (0.9‐1.8)	.15
LVDDn	1.5 ± 0.1	1.5 ± 0.2	.72
LVDSn	**1.0 (0.6–1.6)**	**1.0 (0.6‐1.3)**	**.03**
FS (%)	**27 ± 9**	**30 ± 7**	**.002**
EPSSn	**1.92 (0.47‐7.1)**	**1.3 (0.41‐2.32)**	**.001**
E‐wave velocity (cm/s)	**59 ± 15**	**66 ± 13**	**.009**
A‐wave velocity (cm/s)	**47 ± 14**	**54 ± 13**	**.002**
MPI	0.56 ± 0.24	0.55 ± 0.16	.82
EDVi (mL/m^2^)	**62 (28–101)**	**60 (32‐87)**	**.026**
ESVi (mL/m^2^)	34 ± 12	29 ± 10	.10
EF (%)	48 ± 11	51 ± 10	.35

*Note:* Normally and nonnormally distributed data are expressed as mean ± standard deviation and median and range (minimum–maximum), respectively. Values with statistically significant difference are indicated in bold.

Abbreviations: EDVi, end diastolic volume indexed to body surface area; EF, ejection fraction; EPSSn, E‐point‐to‐septal separation normalized to body weight; ESVi, end systolic volume indexed to body surface area; FS, fractional shortening; HR, heart rate; LVDDn, left ventricular diastolic diameter normalized to body weight; LVDSn, left ventricular systolic diameter normalized to body weight; MEA, mean electrical axis; MPI, myocardial performance index; QTc, QT corrected for heart rate, LA/Ao, left atrial to aortic root ratio.

## 4 DISCUSSION

We evaluated the effects of hypothyroidism on cardiac electric activity and mechanical function before and after levothyroxine supplementation. Evaluation included measurement of MPI, an echocardiographic index of the global myocardial function combining both systolic and diastolic cardiac performance. The principal findings were that hypothyroid dogs had mild ECG and echocardiographic changes compared to clinically healthy dogs, and these cardiac changes improved after levothyroxine supplementation. Furthermore, the MPI was not significantly different neither between hypothyroid dogs at the time of diagnosis and control dogs nor in the former dogs before and after approximately 2 months of treatment. Although the effect of thyroid hormones on cardiac function has been investigated previously in dogs,^8^, ^9^ ours is the first study comparing hypothyroid dogs with a control group of clinically healthy dogs.

The differential diagnosis of a dilated and hypokinetic LV is challenging in the dog, and the final diagnosis often can be achieved only by postmortem evaluation of the heart.^15^ Therefore, evaluation of thyroid function has been suggested in the diagnostic evaluation to rule out hypothyroidism‐induced cardiomyopathy.^15^ Based on our results, this assumption likely should be reconsidered. Our findings are similar to those of a recent study focused on the evaluation of the relationship between hypothyroidism and DCM in Doberman Pinschers.^33^ In that study, a cause‐effect relationship between the 2 diseases could not be identified, and the authors stated that hypothyroidism does not play any role in the etiology and progression of DCM.^33^ Similarly, results of our study do not justify considering hypothyroid‐induced cardiac changes as identical to DCM, because the observed echocardiographic changes were too mild to reflect clinically relevant cardiac dysfunction. Some isolated reports of dogs with clinically relevant cardiac dysfunction associated with hypothyroidism have been published previously.^3^, ^4^, ^5^, ^6^ However, these cases were only sporadic compared to the overall prevalence of hypothyroidism in dogs.^34^ It also must be considered that the diagnosis of hypothyroidism in dogs is often challenging, and the risk of misdiagnosing hypothyroidism in dogs with non‐thyroidal illness syndrome is a major concern.^1^, ^33^, ^34^ Dogs enrolled in our study represented a large cohort of hypothyroid dogs that were managed identically. In particular, all dogs underwent a standardized diagnostic protocol and all questionable cases received a rhTSH‐stimulation test to obtain a definitive diagnosis.^22^ Therefore, all dogs in our study had an ascertained diagnosis, but none of them showed clinical signs of cardiac disease nor had clinically relevant cardiac changes detected on ECG and echocardiography.

When systematically considering the measured ECG variables, mean HR was significantly decreased in the hypothyroid dogs at T0 compared to that of the control group, but only 1 hypothyroid dogs was bradycardic. Decreased HR in dogs with spontaneous and experimentally induced hypothyroidism has been reported,^7^, ^9^, ^11^, ^12^ and may be caused by a direct effect of thyroid hormones on the myocardium, decrease in tissue oxygen consumption, or downregulation of β‐adrenergic receptors causing a decreased response to sympathetic stimulation.^35^ The P‐ and R‐wave amplitudes also were significantly decreased in the hypothyroid group compared to those of the control group. In 2 previous studies, low R‐waves were reported in 11 of 19 (58%) and in 24 of 66 (36%) of hypothyroid dogs.^8^, ^9^ Although the exact cause of low R‐wave amplitude is not known, obesity, decreased myocardial mass, or decreased circulating blood volume associated with hypothyroidism may be responsible for this ECG modification.^35^ Finally, a significantly lower MEA of the QRS between hypothyroid dogs and the control group was noted. Changes in the MEA of the QRS may indicate ventricular chamber enlargement or intraventricular conduction disturbances,^23^ and only 1 previous study reported a slight deviation in the MEA of the QRS in 3 hypothyroid dogs.^11^ However, after reviewing each ECG, we noted that 1 dog had a left axis deviation (ie, MEA = −11°; reference range, +40° to +110°) that normalized after treatment, suggesting a transient intraventricular conduction disturbance that resolved after hormone supplementation. Another dog had a permanent right bundle branch block, with calculated MEA of the QRS of −99°. This case alone might be responsible for the observed shifting of the median MEA of the QRS of the entire hypothyroid group.

Regarding the left‐sided echocardiographic linear measurements and derivate indices, a few hypothyroid dogs had slightly increased LA/Ao and LVDSn (5 dogs and 1 dog, respectively), but the median values were not significantly different compared to those of clinically healthy dogs. Although previous studies documented increased LA and LV dimensions in dogs with hypothyroidism,^5^, ^8^ our results showed that cardiac remodeling with left‐sided cardiac enlargement is not a consistent finding in the overall population of hypothyroid dogs. Moreover, when comparing hypothyroid dogs at T0 and T60, 2 dogs maintained an LA/Ao above the reference limit, and only 1 dog showed an LVDSn value equal to 1.3. These findings suggest that even mild left‐sided cardiac dilatation tends to normalize after appropriate hormone replacement, as observed in other studies.^6^, ^8^ Among the echocardiographic indices of LV systolic function, only EPSSn was significantly increased in hypothyroid dogs compared to control dogs, whereas both EPSSn and FS improved after treatment in dogs with hypothyroidism. These findings may reflect a mild negative inotropic effect induced by the absence of an adequate concentrations of thyroid hormones or, conversely, a positive inotropic effect of hormone replacement.^5^, ^8^, ^36^ The transmitral E‐wave velocity was significantly decreased in hypothyroid dogs at T0 compared to the control group, whereas a significant increase was seen after levothyroxine treatment for both E‐ and A‐wave velocities. Because no significant differences in LA/Ao and LVDDn were observed between the groups, suggesting unchanged LV preload, the observed changes in transmitral blood flow velocities might be explained by slight impairment in LV diastolic function in hypothyroid dogs at T0. On the other hand, increases in E‐ and A‐ wave velocities at T60 more likely may be secondary to an increase in HR than an indication of LV diastolic dysfunction.^37^

No significant difference was found in the mean MPI between hypothyroid dogs at T0 and the control group and between T0 and T60 in hypothyroid dogs. Clinical application of the MPI in dogs includes evaluation of the global ventricular function of the LV, right ventricle or both in Newfoundland dogs with DCM, Boxer dogs with arrhythmogenic right ventricular cardiomyopathy, and in dogs with parvovirus infection, myxomatous mitral valve disease (MMVD), tricuspid regurgitation, or pulmonary arterial hypertension.^38^, ^39^, ^40^, ^41^, ^42^, ^43^, ^44^, ^45^ These studies showed a significant increase in the LV MPI in dogs with symptomatic MMVD compared to control dogs and dogs with asymptomatic MMVD,^40^ as well as in Newfoundland dogs with DCM compared to clinically healthy Newfoundland dogs,^38^ whereas no difference was found in Boxer dogs with or without arrhythmogenic right ventricular cardiomyopathy.^43^, ^44^ Furthermore, an increased LV and right ventricular MPI had negative prognostic value in dogs with parvovirus infection^45^ and in dogs with MMVD,^42^ respectively. Our results suggest that MPI has poor diagnostic usefulness in identifying either the subtle cardiac changes induced by hypothyroidism or the positive changes of levothyroxine supplementation. These findings are in contrast with those in humans with hypothyroidism, in whom measurement of MPI allowed identification of deterioration in cardiac function that was reversible after levothyroxine replacement.^17^, ^20^

Our study had some limitations. First, it was a 2‐center study and data were obtained by 2 independent observers (H.P. and M.B.T.) using different ECG and ultrasound machines, as well as laboratory instruments. However, all of the echocardiographic and ECG data were reviewed by a single board‐certified cardiologist (M.B.T.) and the previously calculated coefficient of variation of the echocardiographic measurements for the 2 observers was low or very low (ie, 5%‐15% and <5%, respectively). Furthermore, diagnostic and therapeutic protocols for hypothyroidism were standardized at the beginning of the study. Second, the echocardiographic assessment of the cardiac function was based only on conventional echocardiographic techniques, and no advanced techniques (eg, tissue Doppler imaging, speckle tracking) were used. These latter techniques are more sensitive to detect subtle changes of systolic or diastolic cardiac function, as recently demonstrated in dogs with other endocrine diseases such as diabetes mellitus and hyperadrenocorticism.^46^, ^47^ Thus, it cannot be completely excluded that any minor changes in cardiac function in the hypothyroid dogs could have been detected using advanced echocardiographic techniques. Finally, the time interval between the visits at T0 and T60 of hypothyroid dogs was not constant, mainly because of owner needs. Thus, a possible effect on the evaluated cardiac function after hormonal replacement cannot be completely excluded.

## 5 CONCLUSIONS

Our results confirmed that hypothyroidism can induce some modifications of the electromechanical cardiac function in affected dogs, but these are usually mild and rapidly reversible after levothyroxine supplementation. None of the hypothyroid dogs showed clinical signs of heart disease as well as echocardiographic features mimicking a DCM phenotype. Finally, MPI, an echocardiographic index of combined systolic and diastolic function, does not seem to be a useful clinical variable to identify cardiac dysfunction in dogs with spontaneous hypothyroidism.

## CONFLICT OF INTEREST DECLARATION

Authors declare no conflict of interest.

## OFF‐LABEL ANTIMICROBIAL DECLARATION

Authors declare no off‐label use of antimicrobials.

## INSTITUTIONAL ANIMAL CARE AND USE COMMITTEE (IACUC) OR OTHER APPROVAL DECLARATION

Authors declare no IACUC or other approval was needed.

## HUMAN ETHICS APPROVAL DECLARATION

Authors declare human ethics approval was not needed for this study.

## References

[1] Scott‐Moncrieff JC . Hypothyroidism In: Feldman EC, Nelson RW, Reusch CE, et al., eds. Canine and Feline Endocrinology. 4th ed. St. Louis: Elsevier; 2015:77‐135.

[2] Scott‐Moncrieff JC . Clinical signs and concurrent diseases of hypothyroidism in dogs and cats. Vet Clin North Am Small Anim Pract. 2007;37:709‐722. 17619007 10.1016/j.cvsm.2007.03.003

[3] Chow B , French A . Conversion of atrial fibrillation after levothyroxine in a dog with hypothyroidism and arterial thromboembolism. J Small Anim Pract. 2014;55:278‐282. 24521196 10.1111/jsap.12184

[4] Gerritsen RJ , van den Brom WE , Stokhof AA . Relationship between atrial fibrillation and primary hypothyroidism in the dog. Vet Q. 1996;18:49‐51. 8792593 10.1080/01652176.1996.9694614

[5] Flood JA , Hoover JP . Improvement in myocardial dysfunction in a hypothyroid dog. Can Vet J. 2009;50:828‐834. 19881920 PMC2711467

[6] Phillips DE , Harkin KR . Hypothyroidism and myocardial failure in two great Danes. J Am Anim Hosp Assoc. 2003;39:133‐137. 12617541 10.5326/0390133

[7] Swinney GR , Malik R . Intermittent heart block in a lethargic dog. Aust Vet J. 1998;76(663):667‐668. 10.1111/j.1751-0813.1998.tb12277.x 9830564

[8] Panciera DL . A retrospective study of 66 cases of canine hypothyroidism. J Am Vet Med Assoc. 1994;204:761‐767. 8175472

[9] Panciera DL . An echocardiographic and electrocardiographic study of cardiovascular function in hypothyroid dogs. J Am Vet Med Assoc. 1994;205:996‐1000. 7852177

[10] Wysoke JM , Van Heerden J . Electrocardiographic changes associated with altered thyroid function in two dogs. J S Afr Vet Assoc. 1990;61:130‐132. 2287002

[11] Nijhuis AH , Stokhof AA , Huisman GH , Rijnberk A . ECG changes in dogs with hypothyroidism. Tijdschr Diergeneeskd. 1978;103:736‐741. 675644

[12] Goel Brij G , Catherine MD , Hanson S , Jaok BS , Han MD . A‐V conduction in hyper‐ and hypothyroid dogs. Am Heart J. 1972;83:504‐511. 5041838 10.1016/0002-8703(72)90041-5

[13] Pasławska U , Noszczyk‐Nowak A , Kungl K , et al. Thyroid hormones concentrations and ECG picture in the dog. Pol J Vet Sci. 2006;9:253‐257. 17203744

[14] Stephan I , Nolte I , Hoppen HO . The effect of hypothyroidism on cardiac function in dogs. Dtsch Tierarztl Wochenschr. 2003;110:231‐239. 12866255

[15] Dukes‐McEwan J , Borgarelli M , Tidholm A , Vollmar AC , Häggström J , ESVC Taskforce for Canine Dilated Cardiomyopathy . Proposed guidelines for the diagnosis of canine idiopathic dilated cardiomyopathy. J Vet Cardiol. 2003;5:7‐19. 10.1016/S1760-2734(06)70047-9 19081360

[16] Miller CW , Boon JA , Soderburg SA , et al. Echocardiographic assessment of cardiac function in beagles with experimentally produced hypothyroidism (abstract). J Ultrasound Med. 1984;3:157.

[17] Doin FLC , Borges MDR , Campos O , et al. Effect of central hypothyroidism on Doppler‐derived myocardial performance index. J Am Soc Echocardiogr. 2004;17:622‐629. 15163932 10.1016/j.echo.2004.03.010

[18] Tei C , Ling LH , Hodge DO , et al. New index of combined systolic and diastolic myocardial performance: a simple and reproducible measure of cardiac function‐study in normals and dilated cardiomyopathy. J Cardiol. 1995;26:357‐366. 8558414

[19] Tei C . New non‐invasive index for combined systolic and diastolic ventricular function. J Cardiol. 1995;26:135‐136. 7674144

[20] Yazici M , Gorgulu S , Sertbas Y , et al. Effects of thyroxin therapy on cardiac function in patients with subclinical hypothyroidism: index of myocardial performance in the evaluation of left ventricular function. Int J Cardiol. 2004;95:135‐143. 15193811 10.1016/j.ijcard.2003.05.015

[21] Iversen L , Jensen AL , Høier R , Aaes H . Biological variation of canine serum thyrotropin (TSH) concentration. Vet Clin Pathol. 1999;28:16‐19. 12075532 10.1111/j.1939-165x.1999.tb01036.x

[22] Boretti FS , Sieber‐Ruckstuhl NS , Favrot C , Lutz H , Hofmann‐Lehmann R , Reusch CE . Evaluation of recombinant human thyroid‐stimulating hormone to test thyroid function in dogs suspected of having hypothyroidism. Am J Vet Res. 2006;67:2012‐2016. 17144802 10.2460/ajvr.67.12.2012

[23] Willis R . Sinus rhythms In: Willis R, Oliveira P, Mavropoulou A, eds. Guide to Canine and Feline Electrocardiography. 1st ed. Oxford: John Wiley & Sons; 2018:58‐66.

[24] Matsunaga T , Mitsui T , Harada T , Inokuma M , Murano H , Shibutani Y . QT corrected for heart rate and relation between QT and RR intervals in beagle dogs. J Pharmacol Toxicol Methods. 1997;38:201‐209. 9566444 10.1016/s1056-8719(97)00098-1

[25] Santilli RA , Perego M . Genesi e interpretazione delle onde elettrocardiografiche In: Santilli RA, Perego M, eds. Elettrocardiografia del cane e del Gatto. 1st ed. Milano: Elsevier; 2009:29‐55.

[26] Thomas WP , Gaber CE , Jacobs GJ , et al. Recommendations for standards in transthoracic two‐dimensional echocardiography in the dog and cat. J Vet Intern Med. 1993;7:247‐252. 8246215 10.1111/j.1939-1676.1993.tb01015.x

[27] Sahn DJ , DeMaria A , Kisslo J , Weyman A . Recommendations regarding quantitation in M‐mode echocardiography: results of a survey of echocardiographic measurements. Circulation. 1978;58:1072‐1083. 709763 10.1161/01.cir.58.6.1072

[28] Cornell CC , Kittleson MD , Della Torre P , et al. Allometric scaling of M‐mode cardiac measurements in normal adult dogs. J Vet Intern Med. 2004;18:311‐321. 15188817 10.1892/0891-6640(2004)18<311:asomcm>2.0.co;2

[29] Kirberger RM . Mitral valve E point to ventricular septal separation in the dog. J S Afr Vet Assoc. 1991;62:163‐166. 1770491

[30] Morrison SA , Moise NS , Scarlett J , Mohammed H , Yeager AE . Effect of breed and body weight on echocardiographic values in four breeds of dogs of different somatotype. J Vet Intern Med. 1992;6:220‐224. 1522552 10.1111/j.1939-1676.1992.tb00342.x

[31] Rishniw M , Erb HN . Evaluation of four 2‐dimensional echocardiographic methods of assessing left atrial size in dogs. J Vet Intern Med. 2000;14:429‐435. 10935894 10.1892/0891-6640(2000)014<0429:eofemo>2.3.co;2

[32] Bruch C , Schmermund A , Marin D , et al. Tei‐index in patients with mild‐to‐moderate congestive heart failure. Eur Heart J. 2000;21:1888‐1895. 11052862 10.1053/euhj.2000.2246

[33] Beier P , Reese S , Holler PJ , et al. The role of hypothyroidism in the etiology and progression of dilated cardiomyopathy in Doberman Pinschers. J Vet Intern Med. 2015;29:141‐149. 25306963 10.1111/jvim.12476 PMC4858054

[34] Mooney CT . Canine hypothyroidism In: Ettinger SJ, Feldman EC, Còté E, eds. Textbook of Veterinary Internal Medicine. 8th ed. St. Louis: Elsevier; 2017:1731‐1742.

[35] Panciera DL . Conditions associated with canine hypothyroidism. Vet Clin North Am Small Anim Pract. 2001;31:935‐950. 11570133

[36] Holler PJ , Wess G . Sphericity index and E‐point‐to‐septal‐separation (EPSS) to diagnose dilated cardiomyopathy in Doberman pinschers. J Vet Intern Med. 2014;28:123‐129. 24428318 10.1111/jvim.12242 PMC4895524

[37] Schober KE , Fuentes VL . Effects of age, body weight, and heart rate on transmitral and pulmonary venous flow in clinically normal dogs. Am J Vet Res. 2001;62:1447‐1454. 11560276 10.2460/ajvr.2001.62.1447

[38] Lee BH , Dukes‐McEwan J , French AT , Corcoran BM . Evaluation of a novel Doppler index of combined systolic and diastolic myocardial performance in Newfoundland dogs with familial prevalence of dilated cardiomyopathy. Vet Radiol Ultrasound. 2002;43:154‐165. 11954811 10.1111/j.1740-8261.2002.tb01663.x

[39] Teshima K , Asano K , Iwanaga K , et al. Evaluation of right ventricular Tei index (index of myocardial performance) in healthy dogs and dogs with tricuspid regurgitation. J Vet Med Sci. 2006;68:1307‐1313. 17213699 10.1292/jvms.68.1307

[40] Teshima K , Asano K , Iwanaga K , et al. Evaluation of left ventricular Tei index (index of myocardial performance) in healthy dogs and dogs with mitral regurgitation. J Vet Med Sci. 2007;69:117‐123. 17339754 10.1292/jvms.69.117

[41] Paradies P , Spagnolo PP , Amato ME , Pulpito D , Sasanelli M . Doppler echocardiographic evidence of pulmonary hypertension in dogs: a retrospective clinical investigation. Vet Res Commun. 2014;38:63‐71. 24414341 10.1007/s11259-013-9588-4

[42] Nakamura K , Morita T , Osuga T , et al. Prognostic value of right ventricular Tei index in dogs with myxomatous mitral valvular heart disease. J Vet Intern Med. 2016;30:69‐75. 26789419 10.1111/jvim.13820 PMC4913668

[43] Baumwart RD , Meurs KM , Bonagura JD . Tei index of myocardial performance applied to the right ventricle in normal dogs. J Vet Intern Med. 2005;19:828‐832. 16355676 10.1892/0891-6640(2005)19[828:tiompa]2.0.co;2

[44] Baumwart RD , Meurs KM . An index of myocardial performance applied to the right ventricle of boxers with arrhythmogenic right ventricular cardiomyopathy. Am J Vet Res. 2008;69:1029‐1033. 18672967 10.2460/ajvr.69.8.1029

[45] Kocaturk M , Martinez S , Eralp O , Tvarijonaviciute A , Ceron J , Yilmaz Z . Tei index (myocardial performance index) and cardiac biomarkers in dogs with parvoviral enteritis. Res Vet Sci. 2012;92:24‐29. 21074228 10.1016/j.rvsc.2010.10.018

[46] Kim YH , Kim JH , Park C . Evaluation of tissue Doppler ultrasonographic and strain imaging for assessment of myocardial dysfunction in dogs with type 1 diabetes mellitus. Am J Vet Res. 2018;79:1035‐1043. 30256147 10.2460/ajvr.79.10.1035

[47] Chen HY , Lien YH , Huang HP . Assessment of left ventricular function by two dimensional speckle‐tracking echocardiography in small breed dogs with hyperadrenocorticism. Acta Vet Scand. 2014;56:88. 25551792 10.1186/s13028-014-0088-5 PMC4300024

